# Effectiveness of simplified ART regimens in older adults living with HIV: real-world evidence from a Brazilian cohort

**DOI:** 10.1590/1414-431X2025e14994

**Published:** 2026-01-09

**Authors:** P.V. Stanicki, K.I. Tasca, W.R. Zimmerman, A.N. Barbosa

**Affiliations:** 1 Departamento de Infectologia, Faculdade de Medicina de Botucatu, Universidade Estadual Paulista (UNESP), Botucatu, SP, Brasil; 2 Center for Global Health, Science, and Security, Georgetown University, Washington DC, USA

**Keywords:** HIV, Aging, Antiretroviral therapy, Simplified regimens

## Abstract

Antiretroviral therapy (ART) has transformed HIV from a life-limiting infection into a manageable chronic condition, shifting attention toward the emerging challenges faced by the growing population of adults aged 50 years or older living with HIV. This observational cohort study tracked 1,018 individuals treated in the medical system in Brazil, a middle-income country, to better understand the effectiveness of traditional and simplified ART regimens in older adults living with HIV (OALH), a frequently underrepresented group in clinical studies. Older adults, those aged 50 and above, living with HIV achieved significantly higher rates of undetectable viral load (89.4 *vs* 83.2%, P<0.006) and fewer cases of virological failure, defined as HIV-RNA >500 copies/mL (2.5 *vs* 10.1%; P<0.0001) than younger adults aged 18-49, and demonstrated superior immune recovery through significantly greater CD4+ T-cell counts (P=0.0012). The multivariate analysis found that improved clinical outcomes (undetectable viral load) were most highly correlated with the simplified treatment regime and more years of treatment duration. These findings highlight the value of simplified ART regimens and sustained treatment duration, which were observed to be more frequent in OALH.

## Introduction

Antiretroviral therapy (ART) has revolutionized the treatment of HIV, transforming it into a chronic, manageable condition with a life expectancy now comparable to the general population for those with access to care. Global estimates indicate that the proportion of people living with HIV aged 50 years or older increased from 8.5% in 2000 to 21.4% in 2020, with a similar trend in Latin America (rising from 12.1 to 27.4%) (1).

Older adults (aged 50 years or older) living with HIV (OALH) face a complex interplay of factors that can impede sustained virological suppression. These include chronic inflammation, accelerated aging, and a high prevalence of comorbidities, all of which are further compounded by polypharmacy, drug-drug interactions, and stigma (2,3).

In recent years, simplified antiretroviral regimens, typically composed of two active agents instead of the conventional three, have gained increasing attention as potential solutions to these emerging complexities (4). Clinical trials such TANGO (2020) and SWORD-1 and SWORD-2 (2018) have demonstrated that two-drug regimens can achieve similar virological suppression while reducing adverse events, suggesting potential advantages for OALH who may already grapple with multiple health conditions and higher rates of polypharmacy (5,6). Although these are promising findings, most available data originate from high-income countries, and evidence on the effectiveness and long-term viability of these simplified therapies in middle-income settings remains sparse (5).

Considering this demographic shift and the paucity of data from middle-income settings, this observational cohort study evaluated the real-world effectiveness of simplified two-drug regimens in OALH by directly comparing their virological and immunological outcomes with those achieved using conventional three-drug antiretroviral therapy, under the hypothesis that simplified ART regimens are associated with superior virological and immunologic outcomes, especially in OALH.

## Material and Methods

This observational cohort study initially included 1,094 people living with HIV (PLHIV) aged 18 years and above receiving ART at the “Domingos Alves Meira” Specialized Infectious Diseases Outpatient Service (SAEI-DAM), Clinical Hospital Complex of the Botucatu Medical School, São Paulo State Universtiy, between January 2020 and July 2022. These patients had a confirmed HIV diagnosis, initiated ART before or during January 2021, consistently collected medication through July 2023, attended scheduled visits, and had at least 6 months of ART. Of those, 76 were excluded because they did not adhere to follow-ups or had missing data. Non-adherence was defined as missing two or more consecutive scheduled clinic visits or medication pick-ups. No sample size calculation was performed, as we analyzed the entire eligible population attending our service to maximize generalizability.

Participants were categorized into two groups: Group 1 (G1-OALH): PLHIV aged ≥50 years and Group 2 (G2, younger adults (YALH); control group): PLHIV aged 18-49 years. Clinical and demographic data, including ART regimens, duration of treatment, CD4 T-cell counts, and viral suppression rates were extracted from electronic medical records. ART duration was obtained from the initial prescription records in electronic medical files. We used the most recent available value (last testing) within the study period for both viral load and CD4 count. All variables were entered into a standardized Excel database and coded using a predefined codebook. To ensure accuracy, the authors performed a quality check by reviewing 25% of all records.

ART regimens were categorized as either simplified dual therapy regimens or conventional three-drug regimens. Simplified regimens included lamivudine + dolutegravir (3TC+DTG), lamivudine + ritonavir-boosted darunavir (3TC+DRV/r), and dolutegravir + darunavir/ritonavir (DTG+DRV/r). Conventional regimens were primarily based on tenofovir + lamivudine + dolutegravir (TDF+3TC+DTG). Additional combinations observed in the cohort included TDF/3TC/efavirenz (EFZ), TDF/3TC + atazanavir/ritonavir (ATV/r), TDF/3TC+DTG+DRV/r, and TDF/3TC + nevirapine (NVP), among others. ART effectiveness was defined as sustained viral suppression indicated by HIV-RNA <40 copies/mL, while virological failure was defined as HIV-RNA >500 copies/mL.

Statistical analyses included chi-squared tests for categorical variables and *t*-tests for continuous variables to compare groups. A logistic regression model was used to assess predictors associated with therapeutic success. The dependent variable was undetectable viral load, and the independent variables were age group, sex, ART duration, and ART treatment. Odds ratios and 95% confidence intervals were calculated. All analyses were performed in SAS for Windows version 9.4, and a P-value <0.05 was considered statistically significant.

The study was approved by the Research Ethics Committee of Botucatu Medical School (CAAE: 61694222.1.0000.5411) and complied with the principles of the Declaration of Helsinki.

## Results

Of the included participants, 56.58% (n=576) were aged 50 years or older and 43.42% (n=442) were younger than 50. In terms of treatment duration, 57.33% (n=583) had received ART for 0-10 years, whereas 42.67% (n=434) had been on therapy for 11 years or more. The majority of the cohort was male (66.4%, n=676), with females constituting 33.6% (n=342). These data are shown in Table 1.

**Table 1:** Comparative analysis of virological and immunological outcomes by age group (18-49 *vs* ≥50 years) among people living with HIV receiving ART at SAEI-DAM, Botucatu-SP, Brazil (January 2020-July 2022).

Variable	Total population (n=1018)	Age 18-49 (n=576)	Age ≥50 (n=442)	P-value
Male sex (%)	676 (66.4%)	65.5%	67.9%	0.432
ART duration ≥11 years (%)	434 (42.67%)	39.2%	47.1%	0.010
CD4+ T <350 cells/mm^3^ (%)	112 (11.0%)	13.3%	7.0%	0.0012
Undetectable viral load (<40 copies/mL)	877 (86.15%)	83.3%	89.8%	<0.0001
Residual viremia (40-500 copies/mL)	72 (7.07%)	6.6%	7.7%	0.538
Virological failure (>500 copies/mL)	69 (6.78%)	10.1%	2.5%	<0.0001
TARV: TDF/3TC/DTG regimen (%)	419 (41.16%)	41.7%	40.2%	0.610
Simplified (2-drug) regimen (%)	330 (32.42%)	28.3%	37.8%	0.003

ART: antiretroviral therapy; CD4: CD4+ T lymphocyte count; TDF: tenofovir; 3TC: lamivudine; DTG: dolutegravir. *P-values were derived from chi-squared tests. To calculate the percentages, we assumed the fraction relative to the number of corresponding participants in the same column.

OALH exhibited a higher rate of virological suppression (89.8%) compared to YALH (83.3%), alongside fewer virological failures (HIV-RNA >500 copies/mL, P<0.05). Considering the entire cohort, the immune status profile revealed that 11% (n=112) had CD4 T-cell counts <350 cells/mm^3^, whereas 89% (n=906) exceeded 350 cells/mm^3^. Regarding antiretroviral regimens, 41.16% (n=419) of all patients were prescribed TDF/3TC plus DTG, 24.66% (n=251) received 3TC+DTG, 9.72% (n=99) were on TDF/3TC plus DRV, 7.76% (n=79) took 3TC plus DRV, and 16.7% (n=170) followed other ART combinations. Viral load measurements across the entire sample revealed that 86.15% (n=877) had undetectable levels (<40 copies/mL), 7.07% (n=72) displayed low-level viremia (50-500 copies/mL), and 6.78% (n=69) had HIV-RNA counts above 500 copies/mL. Simplified two-drug regimens, such as 3TC+DTG or 3TC+DRV/r, were more frequently utilized in OALH (7%) *vs* 18-49 years (13%; P=0.003) and were associated with the best immune recovery, showing the highest CD4 counts (P=0.0012) (Table 1).

Among OALH, there was a significant difference: the highest CD4 counts were observed in those taking regimens 3TC+DTG and 3TC+DRV/r (P<0.0001). In individuals under 50 years old, no significant difference was observed between regimens in relation to CD4 counts (data not shown). However, there was also no significant difference between the groups regarding persistent low viremia, defined as HIV-RNA levels between 50 and 500 copies/mL. Notably, age group (≥50 *vs* 18-49), sex, and baseline CD4 count were not significantly associated with virological status in the adjusted model (P<0.29 in all cases).

In the logistic regression, the simplified regimen (3TC+DTG) showed greater chances of sustained viral suppression compared to the other regimens, except for 3TC+DTG/r. Longer time on ART was also a predictor of effectiveness in this cohort, as shown in Table 2. Simplified two-drug regimens were an independent predictor of sustained virological suppression in the entire cohort (OR 2.57; 95%CI: 1.36-4.85; P=0.0006), independent of age group, sex, and baseline CD4 count.

**Table 2:** Logistic regression analysis of predictors of sustained virological suppression (HIV-RNA <40 copies/mL) in people living with HIV receiving ART at SAEI-DAM, Botucatu-SP, Brazil (January 2020-July 2022).

Variable	Odds ratio (OR)	95%CI (Lower)	95%CI (Upper)	P-value
Age group (18-49 vs ≥50)	0.79	0.51	1.22	0.2954
Sex (male *vs* female)	1.18	0.81	1.74	0.3871
ART duration (≥11 *vs* 0-10 years)	1.57	1.02	2.42	0.0422
TARV 1 *vs* TARV 2	2.57	1.36	4.85	0.0006
TARV 1 *vs* TARV 3	5.32	2.57	10.99	0.0006
TARV 1 *vs* TARV 4	1.66	0.61	4.57	0.2628
TARV 1 *vs* TARV 5	4.20	2.13	8.26	0.0075

TARV 1 (reference group) refers to one NRTI plus dolutegravir (3TC+DTG). TARV 2: includes two NRTIs plus dolutegravir (TDF/3TC+DTG); TARV 3: two NRTIs plus ritonavir-boosted darunavir (DRV/r); TARV 4: one NRTI plus DRV/r (3TC+DRV/r); TARV 5: other regimens (e.g., TDF/3TC/EFZ, TDF/3TC+ATV/r, combinations with nevirapine (NVP), or multi-drug regimens).

## Discussion

Patients treated with two-drug regimens for a longer period exhibited higher virological suppression rates and fewer virological failures, and these features were more common among OALH. These findings are consistent with the TANGO clinical trial (2020) and SWORD-1 and SWORD-2 (2018) studies, which reported non-inferior suppression and reduced adverse events for dual therapy compared to traditional three-drug regimens (5,6). Additionally, these results support prior research linking medication simplification to other benefits such as improvements in medication tolerability, patient satisfaction, overall quality of life, and reduced polypharmacy, which make it easier to manage drug-drug interactions (7,8).

Emerging evidence suggests that dual regimens combining DTG and 3TC, which do not include TDF, may offer comparable virological suppression to conventional triple therapies while also favoring immune restoration. Studies have reported meaningful CD4+ T-cell recovery in both naive patients and those who switch from other regimens after achieving viral suppression. For instance, one study documented a mean CD4 increase of nearly 300 cells/μL after 96 weeks of DTG/3TC, mirroring outcomes seen with standard first-line ART (9). Another study found that, among individuals with high baseline viral loads, the DTG/3TC combination outperformed TDF-based regimens in terms of CD4 recovery (10). Additionally, immunologic benefits appeared to extend beyond CD4 count - no significant difference was observed in CD4/CD8 ratio restoration between dual and triple-drug strategies, reinforcing the notion of robust immune reconstitution (11). Collectively, these findings point to simplified regimens as a viable approach not only for maintaining viral control, but also for supporting immune health, particularly in populations affected by comorbidities or cumulative ART exposure. While those trials were conducted in general adult populations, our findings contribute with additional insight into the effectiveness of simplified regimens specifically among OALH, a group often underrepresented in clinical studies.

This study contributes with real-world data from a middle-income country, underscoring the feasibility and effectiveness of simplified regimens in diverse clinical environments. Notably, our cohort's OALH maintained favorable immune status despite the higher prevalence of comorbidities, suggesting that a reduced pill burden and lower toxicity contributed to the observed success. In addition, several variables, which could have additional explanatory power, were not evaluated in this study. Most notably, because our study relied on clinical records of appointment attendance and medication pick-ups, we could not measure at-home adherence and antiretroviral plasma concentrations, or directly assess adherence through pharmacokinetic data, which limited our ability to confirm drug intake or evaluate potential differences in drug metabolism and bioavailability between older and younger adults. It is possible that part of the predictive effect attributed to treatment strategy is due to the fact that patients’ behavioral patterns change as they age; other studies have indicated that OALH often adhere more strictly to treatment regimens than younger adults (8), potentially due to increased health awareness or more frequent clinical monitoring for age-related comorbidities. These additional effects may contribute to the improved outcomes we observed in the OALH cohort.

As a retrospective cohort study conducted at a single center, our findings may not be generalizable to all middle-income settings or to the broader Brazilian population of PLHIV, and results of some of the sub-group analyses must be interpreted with caution due to the small sample size and limited statistical power. As always, errors within electronic medical records, which may affect data accuracy and completeness, cannot be ruled out.

In conclusion, the higher virological suppression rates and reduced treatment failure rates indicate that simplified two-drug regimens could be a valuable strategy especially in the aging population. Future investigations should evaluate the long-term compliance of dual therapy, its cost-effectiveness, and its impact on patient-reported outcomes, particularly in settings with limited resources. Further studies are also needed to clarify whether factors such as greater adherence, reduced toxicity, or both are the primary drivers of improved outcomes in patients receiving simplified ART.

## Data Availability

The data that support the findings of this study are available from the corresponding author on reasonable request.
